# Supplementary material for: Asynchronous magnetic resonance elastography: Shear wave speed reconstruction using noise correlation of incoherent waves
Source: Magn Reson Med. 2022 Oct 27;89(3):990–1001. doi: 10.1002/mrm.29502 (PMC9792433; doi:10.1002/mrm.29502)
Supplement: Supplementary file 1 — DATA S1 MATLAB code used to generate the numerical simulations of Figure 1 [file MRM-89-990-s002.zip › k-Wave/helpfiles/kspaceFirstOrder3DC.html]

kspaceFirstOrder3DC :: Functions (k-Wave)


# kspaceFirstOrder3DC

3D time-domain simulation of wave propagation using C++ code.

## Syntax

See `kspaceFirstOrder3D`.

## Description

`kspaceFirstOrder3DC` provides a blind interface to the C++ version of `kspaceFirstOrder3D` (called `kspaceFirstOrder-OMP`). Note, the C++ code does not support all input options, and all display options are ignored (only command line outputs are given). See the k-Wave user manual for more information.

The function works by appending the optional input `'SaveToDisk'` to the user inputs and then calling `kspaceFirstOrder3D` to save the input files to disk. The contents of `sensor.record` (if set) are parsed as input flags, and the C++ code is run using the `system` command. The output files are then automatically loaded from disk and returned in the same fashion as `kspaceFirstOrder3D`. The input and output files are saved to the temporary directory native to the operating system, and are deleted after the function runs.

This function is not recommended for large simulations, as the input variables will reside twice in main memory (once in MATLAB, and once in C++). For large simulations, the C++ code should be called outside of MATLAB. Again, see the k-Wave manual for more information.

This function requires the C++ binary/executable of `kspaceFirstOrder-OMP` to be downloaded from www.k-wave.org/download.php and placed in the "binaries" directory of the k-Wave toolbox (the same binary is used for simulations in 2D, 3D, and axisymmetric coordinates). Alternatively, the name and location of the binary can be specified using the optional input parameters `'BinaryName'` and `'BinariesPath'`.

## Optional Inputs

Optional 'string', value pairs that may be used to modify the default computational settings.

| Input | Valid Settings | Default | Description |
| --- | --- | --- | --- |
| `'BinaryName'` | *(string)* | `'kspaceFirstOrder-OMP'` on linux and `'kspaceFirstOrder-OMP.exe'` on windows | Name of the binary file. |
| `'BinaryPath'` | *(string)* | `'binaries/'` | Path of the binary file. |
| `'DataName'` | *(string)* | `'kwave_[input/output]_data_[date].h5'` | Prefix used to generate a custom name for the input and output data files (this is appended with `'_input.h5' and '_output.h5').` |
| `'DataPath'` | *(string)* | path returned by `tempdir` | Location of the folder where the input and output HDF5 files should be stored. |
| `'DeleteData'` | *(Boolean scalar)* | `true` | Boolean controlling whether the input and output HDF5 files should be deleted after running the simulation. |
| `'DeviceNum'` | *(integer scalar)* | first free device | GPU device number if running GPU binary, where 0 is first device, 1 is second device, etc. |
| `'FunctionName'` | *(string)* | `kspaceFirstOrder3D` | Name of the k-Wave MATLAB function used to generate the input file using the `'SaveToDisk'` option. |
| `'NumThreads'` | *(integer scalar)* | number of cores | Number of threads used. Note, setting the number of threads greater than the number of cores will decrease performance. |
| `'ThreadBinding'` | *(integer scalar)* `0 = SPREAD 1 = CLOSE` | `0` | Setting for the thread binding policy (sets the environmental variable `OMP_PROG_BIND`). Note, the domain for thread migration is always set to `OMP_PLACES=cores`. Setting `'ThreadBinding', 1` can improve performance when running two instances of MATLAB to run two simulations. In this case, `'NumThreads'` for each simulation should be set to half the maximum number. |
| `'SystemCall'` | *(string)* | `''` | String containing additional system calls that are made before calling the k-Wave binary to run the simulation. |

## See Also

`kspaceFirstOrder2DC`, `kspaceFirstOrder3D`, `kspaceFirstOrder3DG`, `kspaceFirstOrderASC`
